# SNP genotyping reveals genetic diversity between cultivated landraces and contemporary varieties of tomato

**DOI:** 10.1186/1471-2164-14-835

**Published:** 2013-11-27

**Authors:** Giandomenico Corrado, Pietro Piffanelli, Martina Caramante, Mariangela Coppola, Rosa Rao

**Affiliations:** Dipartimento di Agraria, Università degli Studi di Napoli Federico II, via Università 100, 80055 Portici, NA Italy; Parco Tecnologico Padano, via Einstein - Loc. Cascina Codazza, 26900 Lodi, MI Italy

**Keywords:** Population structure, Genetic differentiation, Selection, Germplasm, *Solanum lycopersicum*

## Abstract

**Background:**

The tomato (*Solanum lycopersium* L.) is the most widely grown vegetable in the world. It was domesticated in Latin America and Italy and Spain are considered secondary centers of diversification. This food crop has experienced severe genetic bottlenecks and modern breeding activities have been characterized by trait introgression from wild species and divergence in different market classes.

**Results:**

With the aim to examine patterns of polymorphism, characterize population structure and identify putative loci under positive selection, we genotyped 214 tomato accessions (which include cultivated landraces, commercial varieties and wild relatives) using a custom-made Illumina SNP-panel. Most of the 175 successfully scored SNP loci were found to be polymorphic. Population structure analysis and estimates of genetic differentiation indicated that landraces constitute distinct sub-populations. Furthermore, contemporary varieties could be separated in groups (processing, fresh and cherry) that are consistent with the recent breeding aimed at market-class specialization. In addition, at the 95% confidence level, we identified 30, 34 and 37 loci under positive selection between landraces and each of the groups of commercial variety (cherry, processing and fresh market, respectively). Their number and genomic locations imply the presence of some extended regions with high genetic variation between landraces and contemporary varieties.

**Conclusions:**

Our work provides knowledge concerning the level and distribution of genetic variation within cultivated tomato landraces and increases our understanding of the genetic subdivision of contemporary varieties. The data indicate that adaptation and selection have led to a genomic signature in cultivated landraces and that the subpopulation structure of contemporary varieties is shaped by directed breeding and largely of recent origin. The genomic characterization presented here is an essential step towards a future exploitation of the available tomato genetic resources in research and breeding programs.

**Electronic supplementary material:**

The online version of this article (doi:10.1186/1471-2164-14-835) contains supplementary material, which is available to authorized users.

## Background

The cultivated tomato (*Solanum lycopersicum* L.) was probably domesticated in Mexico from wild species that originated in the Andean region, although other hypotheses have been also put forward [1]. In the XVI century tomato cultivation, which was already well-developed in Central America, was introduced to Europe by Spanish Conquistadors. Although initially viewed as a botanical curiosity, the tomato was almost immediately introduced into the cuisine of different European regions around the Mediterranean basin, starting in Spain and Southern Italy [2, 3]. The tomato later spread to other continents and reached, for instance, North America during the time of the European colonization. At the end of the XIX^th^ century, the tomato varieties were still open pollinated and seeds from the best plants and/or fruits were saved by the farmers every year. Much of the breeding effort took place in the XX^th^ century, when clear distinctions in diverse market classes, such as processing and fresh market, were made [4].

As most of the edible plants, it is likely that the first cultivated tomatoes were directly sampled from wild populations and then improved to obtain a series of types amenable to cultivation. Selection for diverse fruit shapes is one of the distinctive features of the tomato history, along with adaptation to local conditions [1, 4]. Breeding goals have varied and included yield, reduction of production costs, stress resistance, shelf-life and, more recently, taste and nutritional value [1]. Breeding history is associated with apparently contrasting forces. On one hand, tomato suffered different bottlenecks and, when compared with the rich reservoir present in its wild relatives, the amount of genetic variation of the cultivated tomato is considered very limited [5]. On the other hand, since the last century, breeding has been characterized by the introgression of genes for stress resistance from wild species, which has expanded genetic variation [6, 7]. The recent tomato genome sequencing indicated that several chromosomal segments within cultivated varieties are more closely related to *S. pimpinellifolium* than to Heinz 1706. The latter carries introgressions from *S. pimpinellifolium*, which has also been used for the introduction of disease resistance traits, on several chromosomes (4, 9 11 and 12) [8]. Tomato breeding expanded and fixed differences in specific traits. For instance, fruit size, colour and shape present a morphological variety absent in wild species [4], although recent selection may have unintentionally diminished fruit quality in exchange for production traits [9].

Italy and Spain are considered secondary centres of diversification [1, 10, 11]. In Italy, a number of tomatoes with different fruit shapes have been documented since the early days of cultivation [12]. All these types developed into landraces, adapted to the cropping practices and social background in which they were used [1, 12, 13]. It is believed that over the past decades, the cultivated tomato suffered another reduction of diversity due to the disappearance of local varieties [14, 15]. In Italy, despite the good adaptation of landraces to local climatic and soil conditions, the advent of highly productive cultivars after WWII resulted in a very significant decline of their cultivation [13]. Considering the number of documented names and morphological descriptions of home-grown tomato types [16], only a fraction are currently present in local markets [12, 17, 18]. However, cultivated landraces fetch a premium price for their superior flavour and consumers’ affection [19–21].

The analysis of genetic variation in tomato populations has initially focused on differences between wild species and cultivated varieties. More recently, greater attention has been given to the study of the variability present within contemporary varieties. In the tomato inferred subpopulations are associated to breeding history and market classes [6, 22, 23]. It has also been reported that selection for market specialization and for geographic adaptation contributes to the population structure of the tomato cultivars [14, 22].

The major goals of current tomato breeders (e.g.: high quality fruits) require a good understanding and management of the diversity within cultivated genetic resources [24]. Interpreting patterns of genetic variability in cultivated landraces of economically important crops allows breeders to reconsider this trait-reservoir and, eventually, to identify novel alleles or haplotypes to improve productivity, adaptation, quality and nutritional value [25]. To date, much of this germplasm has not been extensively characterized and most of the landraces have yet to be employed in modern plant breeding [26]. Therefore, the study of crop landraces not only provides biological knowledge about its history and value, but is also essential for biodiversity-based breeding [27]. The availability of cost-effective, accurate and fast genotyping assays has made Single Nucleotide Polymorphism (SNP) the most frequently used DNA marker for high-throughput analysis of plants, encouraging the analysis of sequence variation in germplasm collections. In different plant species molecular data have been used to infer the existence of a genetic structure in the collection studied, or to assign individuals to genetically differentiated groups that may be consistent with their ancestry, geographical origin, domestication and/or breeding history [28–30].

In this work we genotyped a wide collection of Italian tomato landraces along with contemporary varieties and wild species. The main goal was to understand whether the human- and environment-driven selection influenced the distribution of genetic variation between contemporary and traditional accessions, leading to the maintenance of a distinct genetic diversity. Furthermore, by using a Fst outlier approach we identified putative loci that can justify the formation of genetically differentiated subpopulations.

## Results

### Genetic diversity

**Figure 1:**
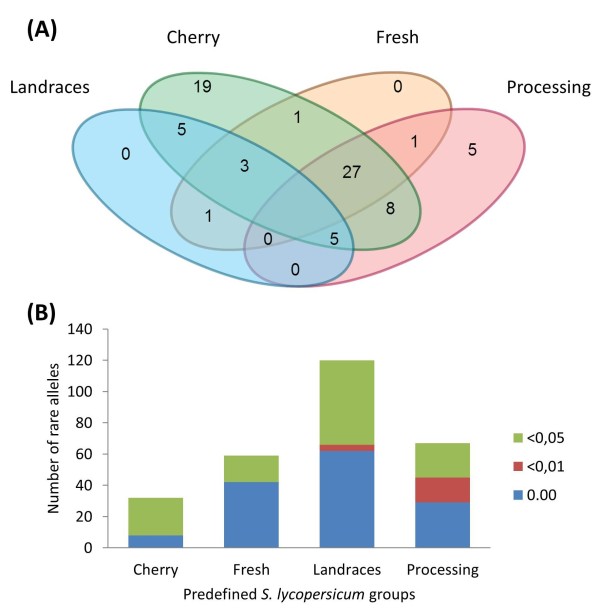
**Allelic distribution in the cultivated** ***S. lycopersicum*** **groups. A**: Distribution of private alleles in the predefined *S. lycopersicum* groups. The Venn diagram illustrates the number of alleles that are exclusive to the various combinations among the four pre-defined groups of cultivated tomatoes. **B**: Number of rare alleles in the pre-defined groups of cultivated tomatoes. For each bar, the height of the colored segments represent the number of alleles that are absent (blue), with a frequency lower than 0.01 (red) or lower than 0.05 (green). See Additional file 5: Table S5 for the name of the alleles.

A total of 177 SNP loci, distributed over the twelve chromosomes, were used to evaluate genetic diversity in 214 genotypes (Additional file 1: Table S1). Two SNPs (SGNU312374-382 and Le004122-27) were removed from subsequent analysis because their flanking sequences map to two locations of the tomato reference genome. The DNA analysis indicated that eleven SNPs were monomorphic. In addition, seventeen SNPs were monomorphic among cultivated *Solanum lycopersicum* genotypes. The summary SNP statistics, which also include Gene Diversity, Heterozygosity and Polymorphic Information Content (PIC), are presented in Additional file 2: Table S2 for all genotypes and Additional file 3: Table S3 for the *S. lycopersicum* varieties and accessions. Allele counts and related frequencies varied among loci (Additional file 4: Table S4) and almost half of the polymorphic loci (49%) presented a major allele frequency higher than 90%. The calculation of the allele frequency for the four predefined *S. lycopersicum* sub-populations (landraces, processing, fresh-market and cherry) allowed the identification of private alleles (i.e.: those occurring in only one population in pairwise comparisons) (Additional file 5: Table S5). Figure 1A shows a Venn diagram indicating the number of alleles that are exclusive to the various group combinations. Overall, the market class cherry presented the highest number of private alleles, while cultivated landraces possess only one private allele when compared to the fresh market varieties. The number of alleles that are absent in each of the pre-defined tomato group was higher for landraces (62), followed by fresh (42), processing (29) and cherry cultivars (8). The number of minor alleles per group (i.e.: those with a frequency lower than 0.05) is presented in Figure 1B.

Table 1 reports the average allelic richness and the average number of alleles. The allelic richness, for both coding and non-coding SNPs, was higher for cherry tomatoes and lower for landraces. Moreover, the highest average number of alleles per locus was found for the non-coding SNPs in the landraces, while for the three market classes of commercial cultivars, there was a slightly higher allelic richness for coding SNPs. The analysis of the inter-groups allelic richness per locus showed low yet statistically significant differences for all but the fresh-processing comparison, corroborating the presence of group-specific differences in the frequency of the analyzed SNPs (Additional file 6: Table S6).

**Table 1:** **Average allelic richness (± standard deviation) and allele per locus in the predefined** ***S. lycopersicum*** **subpopulations**

	Allelic richness			Alleles per locus		
	All	Coding	Non coding	All	Coding	Non coding
Total	1.79 ± 0.41	1.74 ± 0.39	1.70 ± 0.42	1.91	1.87	1.79
Cherry	1.87 ± 0.42	1.83 ± 0.38	1.73 ± 0.45	1.87	1.83	1.73
Fresh	1.67 ± 0.49	1.61 ± 0.48	1.56 ± 0.50	1.67	1.62	1.56
Landraces	1.52 ± 0.46	1.38 ± 0.42	1.42 ± 0.43	1.61	1.48	1.52
Processing	1.69 ± 0.47	1.63 ± 0.44	1.54 ± 0.45	1.75	1.70	1.63

### Population structure

**Figure 2:**
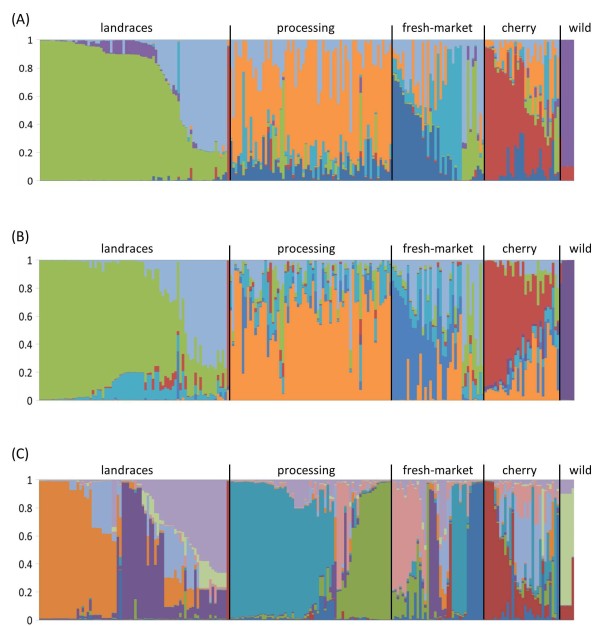
**Estimated population structure of the tomato genotypes.** Each genotype is represented by a horizontal line, which is partitioned into colored segments that represent the estimated membership fractions in the K clusters. **A)** Population structure inferred using the whole SNP dataset for K = 7. **B)** Population structure inferred using the non coding SNPs for K = 7. **C)** Population structure inferred using the coding SNPs for K = 10. See Additional file 7: Figure S1 for the determination of the most informative K value.

We investigated the possible population structure without introducing any a priori classification. The identification of genetically homogeneous groups of plants was performed using an admixture model-based clustering analysis implemented in the software Structure [31]. Three data sets were independently used: the genotyping results with 175 SNPs, 127 non-coding SNPs or 48 coding SNPs. For the whole set of markers, both the Evanno’s test and the non-parametric Kruskal-Wallis analysis indicated that the most informative number of subpopulation (K) was 7 (Additional file 7: Figure S1a and S1b). The Structure analysis provided data for a biological interpretation of the sub-population structure based on the origin and market classes of the contemporary varieties. For this reason, clusters were named according to the a priori group of varieties with the largest membership coefficient. The inferred population structure is presented in Figure 2A and membership coefficients in Additional file 8: Table S7. Landraces grouped together (olive green and light blue). The only exception was the ‘Spongillo’ accession, characterized by small pointed red fruits, which was assigned to the group of contemporary cherry tomatoes. This variety was recently collected from a local farmer and its origin is unknown. The second cluster mainly represents plants with an oxheart (heart-shaped) shaped fruit, such as the ‘Sorrento’ and ‘Cuor di Bue’ types. The contemporary varieties were distributed across more than one group and a distinction could be made among processing, fresh market and cherry tomatoes. The processing varieties were present in one cluster (orange). For fresh market tomatoes a large number of plants appeared to have ancestry in more than one of Structure clusters. Two Structure groups were specific for this market class (dark blue and azure). Admixture with the landrace cluster was evident for cultivars with oxheart fruits (i.e.: ‘Rhodia’, ‘Goldmar’, ‘PS18 3 2693′, ‘Gotico’, ‘Margot’), that often displayed the higher membership coefficient in the group of oxheart landraces. More than half of the cherry varieties showed the highest membership coefficient for a specific cluster (dark red), while the remaining showed admixture, primarily with the processing varieties. Finally, as expected, the tomato’s wild relatives constituted a well separated cluster (purple). The Structure analysis also indicated that among the wild species tested, *S. pimpinellifolium* has the higher admixture with cherry tomatoes [32].

For the non coding SNPs, both the Evanno’s test and the Kruskal Wallis analysis of the log-likelihood variance indicated that the most informative K was 7 (Additional file 7: Figure S1c and S1d). Population structure analysis defined clusters that were associated to a priori tomato type-based groups (Figure 2B). Landraces were divided into two well-defined clusters. Among the contemporary varieties, processing varieties assorted together. Non coding SNPs evidenced the highest level of admixture for the fresh-market tomatoes. Furthermore, the admixture of the cherry varieties with the processing group was more evident. Finally, wild tomatoes grouped separately.

For the coding SNP, the second order rate of change of the likelihood function with respect to K (ΔK) did not show any clear peak at the values tested. The Kruskal-Wallis analysis indicated that the minimum K-value that produced higher likelihood solutions (P < 0.01) was 10, while subsequent K-values had statistically similar solutions (P = 0.492). When the log likelihood score reached a plateau, there was an asymmetric distribution of genotypes and some individuals were strongly assigned to populations, corroborating the presence of a real population structure [31]. At a K-value of 10, a biological interpretation of the assignment was evident (Figure 2C). A division of the genotypes according to the different tomato types was consistent with the previous analysis, but coding SNPs identified further subdivisions. The landraces were partitioned into three sub-groups (orange, purple and liliac). Although plants with different fruit shape were present in each of these groups, approximately half of the plants of the orange group were characterized by having small round/plum fruits. Similarly, the purple group was mostly characterized by plants with cylindrical, elongated ‘San Marzano’ type fruits, and the liliac group by plants with oxheart fruits. Processing varieties were divided in two well separated clusters (green and azure). The fresh market varieties were assigned to different clusters. The majority of the varieties were present in two groups (pink and blue). The others displayed a high membership coefficient with a landraces subpopulation (4 genotype with oxheart fruits; purple) and processing varieties (azure). Two were specific for this market class (pink and blue) however, the other accessions had the higher membership coefficient in the landraces’ group (4 genotypes with oxheart fruits; pink) and processing varieties (azure). Cherry varieties were mostly grouped into two clusters (dark red and light blue) while others displayed a high level of admixture. Wild tomatoes assorted together (light green).

We tested whether the groups inferred by the population structure analysis, or those defined a priori, represent statistically significant subpopulations by pairwise comparison of two measures of differentiation; Fst and Nei’ standard genetic distance (Dst). The results of the two indices (Table 2) were not correlated (P > 0.05, Spearman’s rho test). As expected, higher genetic distances and Fst values were found for the comparison between cultivated material and wild species. The degree of gene differentiation among pre-defined *S. lycopersicum* groups in terms of allele frequencies indicated that landraces represent the most distinct subpopulation compared to each of the contemporary groups considered, as also indicated by the higher Dst values. The three predefined groups of commercial varieties were also significantly different. A minimum genetic distance was determined between fresh market and processing varieties. Both coding and non-coding SNPs were able to support these conclusions (Additional file 9: Table S8) and provided higher values of genetic differentiation and distance. The Structure grouping indicated the presence of a greater subdivision for the landraces and the fresh market groups. These subdivisions were supported by the Fst and Dst values and for each of the two tomato classes the intra-group differentiation and genetic distance were lower when compared to the inter-groups values (Table 2).

**Table 2:** **Estimation of genetic differentiation and distance**

Predefined groups	Cherry	Fresh		Landraces		Processing	Wild
Cherry		0.16**		0.29**		0.12**	0.46 **
Fresh	0.04			0.11**		0.11**	0.60 **
Landraces	0.07	0.03				0.25**	0.69 **
Processing	0.03	0.02		0.06			0.58 **
Wild	0.25	0.34		0.39		0.32	
Structure Groups	C1	F1	F2	L1	L2	P1	W1
C1		0.24**	0.24**	0.37**	0.33**	0.17**	0.49**
F1	0.07		0.11**	0.19**	0.17**	0.12**	0.62**
F2	0.06	0.03		0.24**	0.18**	0.16**	0.59**
L1	0.10	0.04	0.05		0.07**	0.28**	0.69**
L2	0.10	0.03	0.05	0.01		0.21**	0.69**
P1	0.04	0.03	0.04	0.07	0.05		0.57**
W1	0.26	0.36	0.33	0.38	0.39	0.31	

Pairwise estimates of Fst and Nei’s standard genetic distance (Dst) between predefined groups or between groups of tomato accessions as inferred by the Bayesian analysis implemented in the Structure software.

Above the diagonal is the pairwise estimate of Fst, while Dst appears below the diagonal. Global Fst was 0.19 (P < 0.01) within the four tomato groups and 0.26 (P < 0.01) within the Structure groups. The P value for the estimated Fst was calculated using 10,000 permutations (**: P < 0.01).

Coding and non-coding SNPs yielded different subpopulation structures. The analysis of genetic differentiation supported the divisions defined by non-coding SNPs (Additional file 9: Table S8). The additional subdivisions yielded by the coding SNPs were not always statistically supported (Additional file 9: Table S8). The subdivision of landraces into three clusters was significant, as well as the subdivisions of the processing and of the cherry varieties. The analysis of population structure indicated that fresh market tomatoes could be assigned to four groups. However, within them, three subgroups were not statistically different considering the bootstrap analysis of the Fst values. These three groups showed a statistically low or a lack of differentiation also with the wild species, despite that their genetic distance was similar to that of the other pairwise comparisons involving wild species and *S. lycopersicum* varieties. This suggests that the small sample size of the fresh market groups identified by the analysis of population structure may contribute to the lack of a significant genetic differentiation. Finally, pairwise genetic distance and Fst indicated the lack of a significant difference between one fresh market and one processing Structure’s group.

### Loci under selection

**Figure 3:**
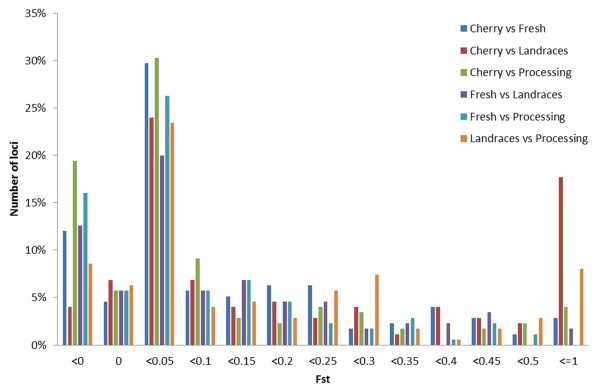
**Distribution of pairwise Fst values among the four cultivated tomato groups.** The percentage of loci for which Fst could not be determined was 15% for C vs F, 15% for C vs L, 13% for C vs P, 28% for F vs L, 24% for F vs P and 22% for L vs P (C: cherry, F: fresh market; L: landraces; P: processing).

**Figure 4:**
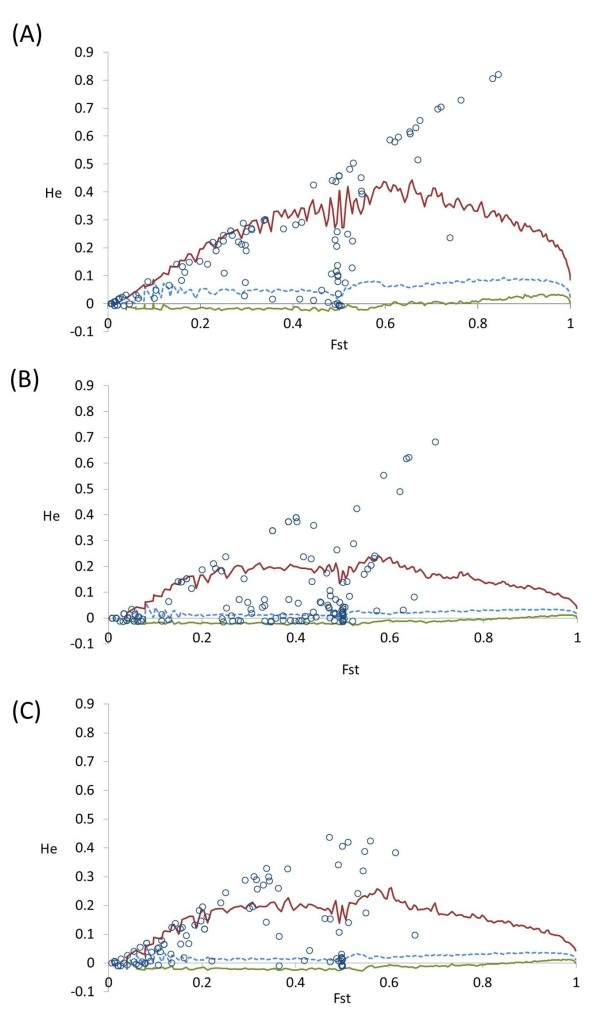
**Plot of Fst against He to identify SNP loci under selection.** Distribution of Fst values as a function of the within-population expected heterozygosity (He). The upper and lower 95% confidence limit for neutrality are indicated by red and green line, respectively. Dashed lines represent the median. **A)** landraces vs processing varieties; **B)** landraces vs cherry varieties; **C)** landraces vs fresh-market varieties.

**Figure 5:**
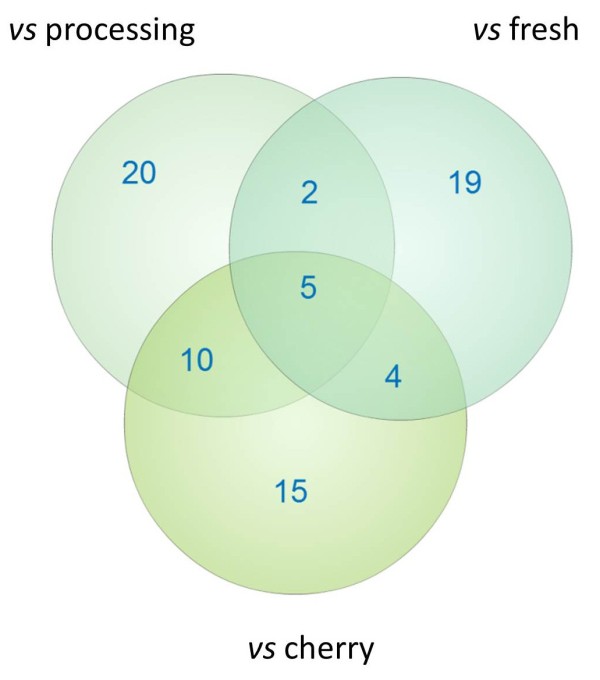
**Number of loci under positive selection between the landraces and the different market classes.** The intersecting portions of the Venn diagram illustrate the number of common loci among the different comparisons.

Locus specific estimates of Fst were calculated to identify genomic regions that have been the target of selection. Wild species were not included in this analysis. A locus-by-locus pairwise Fst comparison between the different tomato classes indicated the presence of substantial variation among loci (Figure 3). A variable percentage of loci, from 4% (cherry vs landraces) to 19% (cherry vs processing) had a negative Fst, reflecting the fact that for these SNPs more variance exists within than across subpopulations. For all comparisons the highest percentage of loci (on average 31%) had Fst values ranging from 0 to 0.05, implying limited variation of allele frequencies between subpopulations. Large differences among pairwise comparisons were found in the number of loci with very high Fst values (>0.5), whose percentage ranged from 18% (cherry vs landraces) to 0 for the fresh vs processing comparison. The percentage of loci that are above the 95% or 99% upper confidence intervals varied little (Additional file 10: Table S9) and implied the presence of outliers in all pairwise comparisons. To statistically identify candidates for loci under selection between landraces and the market classes of commercial varieties we carried out an analysis based on the detection SNPs that had excessively high or low Fst compared to neutral expectations (Figure 4). This method identified 37 SNPs falling outside the 95% confidence boundaries for the landraces vs processing comparison, 34 for the landraces vs fresh market and 30 for the landraces vs cherry (Additional file 11: Table S10). Furthermore, the proportion of coding and non-coding loci under selection was not significantly different from their distribution in the entire dataset (P > 0.05; Pearson’s chi-squared test). Figure 5 illustrates the number of loci that were common or specific in the comparison between landraces varieties and each of the three market classes of contemporary cultivars. Overall, a high proportion of these loci are localized into chromosome 11. Five loci were common among the different comparisons and Table 3 reports their main genetic features. Their functions are consistent with a role in adaptation, as these genes are involved in processes that are vital for plant growth and survival under stressful environmental conditions. It is expected that the majority of the identified loci indicate genomic regions that have been differentiated during selection and breeding, although their detection does not provide indication of the direction of causality. Interestingly, in various cases we found association between two consecutive SNPs of our panel. For instance, the SL10019_376 and the SL10450_71, both localized in chromosome 3, were identified as being putatively under selection in the comparisons between landraces and each of the three classes of contemporary varieties. Furthermore, six consecutive SNPs (SL10240_154, SL20173_496, SL20027_428, SL20181_382, SL10715_489 and SGN-U312814_254) of chromosome 11 were identified as being under selection between the cherry group and landraces.

**Table 3:** **Candidate loci under positive selection that were common among the pairwise comparisons between cultivated landraces and the different market classes of contemporary varieties**

Marker	Chrom.	Exon/intron	Gene name*	Description*	Expected heterozigosity-Fst		
					vs processing	vs fresh market	vs cherry
SL10450_71	3	Exon	Solyc03g114120.2	Ribonuclease III	0.72–0.70	0.14–0.13	0.57–0.24
SL10019_376	3	Intron	Solyc03g113990.2	Uncharacterized conserved protein	0.67–0.66	0.18–0.13	0.56–0.20
SL20017_699	5	Intron	Solyc05g050900.2	Spindle and kinetochore-associated protein 1 homolog	0.53–0.50	0.47–0.44	0.52–0.29
SGN-U313292_417	11	Exon	Solyc11g072190.1	Elongation factor beta-1	0.29–0.29	0.29–0.29	0.40–0.39
SGN-U312814_254	11	Exon	Solyc11g069430.1	Aquaporin 1	0.55–0.40	0.54–0.32	0.62–0.49

*Gene name and description were retrieved from the Solgenomics network (http://solgenomics.net/organism/Solanum_lycopersicum/genome).

## Discussions

Our aim was to investigate population structure and genetic differentiation within the cultivated tomato germplasm and to identify loci that can putatively account for the observed differences. Understanding genetic resources is an important step in order to exploit traits such as nutritional and quality value from cultivated material, especially if it is well adapted to local environments or has not been exposed to modern breeding [25].

Genetic diversity for each of the predefined sub-population was measured using allelic richness, expected heterozygosity, and polymorphic information content. Significant differences among cultivated tomatoes were present considering the allelic richness per locus with the only exception being the fresh vs processing comparison. Landraces have lower allelic richness, a higher number of rare alleles and a lower number of private alleles when compared to contemporary cultivars. Thus, the data suggests that a good portion of the genetic diversity and specific adaptation of the investigated Italian landraces was captured in the founder lines of the contemporary varieties. However, it should be noted that the SNPs employed were selected as polymorphic in contemporary varieties and therefore their use may not be ideal to detect private polymorphisms or rare alleles potentially involved in directional selection of landraces [33]. It is also likely that the very low number of private alleles also reflects the fact that different fruit shapes and plant habits are represented in our landraces collection.

The model-based clustering method for inferring population structure indicated that landraces constitute distinct subpopulations compared to contemporary varieties. This result was evident when considering both non-coding and coding SNPs. Furthermore, our study confirmed that contemporary varieties can be divided into populations that reflect different market classes. All these findings were supported by an analysis of genetic differentiation, which indicated a significant distinction between all tomato types. Our results are consistent with previous studies, which proposed that the genetic differentiation between processing and fresh market varieties mainly reflects breeding for ideotypes related to distinct production systems [22]. In that work, the two processing sub-groups were associated with breeding history in the USA, while sub populations were not discernible in fresh market cultivars and in vintage varieties. We found a subdivision of contemporary cultivars that is associated also to different fruit shapes, as these varieties were separated in three classes (fresh, processing and cherry) rather homogeneous in respect to fruit morphology (round, elongated and cherry, respectively) [34]. Furthermore using the entire SNPs dataset, we did not detect further subdivisions in the processing tomatoes, while the fresh market varieties were assigned to different groups. Irrespective of the type of SNPs employed, fresh market varieties showed the highest degree of population structure, which is coherent with the more competitive breeding activity and diversification of this market class when compared to processing tomatoes [4]. The data also provided evidence for subpopulation structure between cultivated cherry and wild species. Although anticipated [7, 11, 34], a differentiation between cultivated cherry and wild cherry (or landraces) has not always been found [6]. The cherry group showed the highest level of admixture, most likely because several varieties that were assigned to this group lack a clear separation between processing and cherry. For instance, cultivars such as ‘Tomito’, ‘Kikko’, ‘Birba’, ‘Mascalzone’ etc. are improved and sold by breeding companies for both processing and fresh market. Such an explanation is also corroborated by the fact that the high number of loci with negative Fst was present in the cherry vs processing comparison. Overall, considering also the allelic richness and the number of private alleles of the cherry the data indicated that this market class has the highest genetic variation [23, 33]. Our data are consistent with a diverse breeding foundation for the cherry market class.

Selection for fruit shape is considered an important factor responsible for genetic structure in tomato cultivars [35]. It is therefore interesting that the landraces’ subpopulations include a range of fruit shapes (e.g.: elongated, cherry, round, ox heart etc.). Different from other studies, the analysis provided evidence for subpopulations within landraces. A distinction, which was based on fruit shape, was possible for the oxheart type accessions using both coding and non-coding markers.

Overall, our data indicate that the tomato landraces differ from contemporary varieties as the former bears a higher number of minor-alleles (and related allele frequencies) and a stronger population structure, as indicated by the membership coefficient. These features are usually explained considering a strong divergent or directional selection operating on many traits during adaptation to local conditions and practices. Most plant populations are expected to exhibit significant adaptation, especially in the presence of recurrent selection for optimal performance in specific environments [36, 37]. Alternatively, the genetic features of the landraces could be also justified considering the recent tomato history. Breeding of the different market classes has been driven by the common needs of the introgression of traits from wild-species and of lowering the cost of the mechanical practices. However, in this scenario it would be difficult to introduce the population structure of the contemporary varieties that we and others have reported.

We also compared coding and non-coding SNPs. We did not observe large differences in the polymorphism as measured by allelic richness or alleles per locus. As expected, landraces displayed a greater polymorphism in non-coding markers [38, 39] yet contemporary varieties had a greater diversity in coding SNPs. Although all the intronic regions are not necessarily selectively neutral, this may reflect the fact that polymorphism in contemporary varieties essentially derives from breeding efforts. While the analysis of the frequency of minor alleles indicated that selection and adaptation may have changed the frequency of predominant alleles in landraces, the data also suggest that contemporary breeding has increased allelic diversity relative to traditional landraces, especially in coding regions. This hypothesis should be tested by analyzing haplotype structures.

Differences in the ability of markers to discriminate and assign individuals to a subpopulation were not observed for the a priori tomato groups. Irrespective of the type of marker employed, a distinction between landraces and contemporary varieties was well supported. Differences in the number of the optimal number of clusters were present considering the population structure analysis. Coding SNPs distinguished more subpopulations, although not all the groups were different in terms of genetic differentiation. However, the data also suggested that the small number of genotypes in those groups could contribute to the lack of statistically significant differences. The data indicated that the location of the polymorphism within a gene affects the performance for population analysis in the tomato. Although the coding and non coding markers represent different loci, it is reasonably to speculate that the further subdivisions we have observed reflects the fact that genome scans based on coding markers are more likely to detect molecular adaptation linked to genes, although this holds true especially for species with a rapid Linkage Disequilibrium (LD) decay.

The identification of loci that have undergone positive selection is a fundamental step in understanding how populations have adapted to specific environments and agronomic practices. Such studies are increasingly widespread [40, 41] and can also provide insights on the history of the plant species under investigation. Considering that tomato has experienced severe genetic bottlenecks it is difficult to distinguish selective sweeps from the effects of genetic drift due to the bottlenecks themselves. We used an Fst-based statistic to assess if the variation of SNP allele frequencies among populations can identify signatures of selection [41, 42]. If Fst is determined only by genetic drift, the vast majority of the loci should be affected in a similar way [43]. However, we observed the presence of a locus-specific selection pressure in different loci and, in various cases, in linked genetic markers. For instance, the comparison between landraces and cherry tomatoes indicated that some extended chromosomal regions may be under diversifying selection relative to other regions of the genome. Furthermore, considering the number and location of putative loci under selection in studies that mainly compared commercial cultivars [6, 22], the data indicated that various specific regions may differentiate landraces from contemporary varieties.

Although the majority of the loci had a low Fst value in pairwise comparisons, our data showed the presence of genomic regions with high genetic variation between sub-populations. The loci we have identified are of potential interest for plant breeders as they likely contribute to the existing differences between contemporary and local varieties. Considering the LD of the tomato [23], one obstacle is to distinguish genes that are associated from the selected genes themselves. On the other hand, the identification and exclusion of loci under selection is necessary to avoid biased estimates of other genetic parameters such as demographic factors and historical bottlenecks. It is interesting that our results showed that it is possible to efficiently detect a geographical specificity in tomato. Thus, our data imply that it is conceivable to identify markers useful to infer genetic ancestry in cultivated tomato by selecting loci with the highest Fst values and with the ability to yield the largest coefficient of membership for the predefined groups [44]. The loci that can effectively capture variation within populations of interest facilitate candidate gene and fine-structure association studies by allowing for efficient control of population stratification [45]. Besides, their selection is important to identify individuals with greater amounts of admixture so that they can be removed from the breeding pool [46].

## Conclusions

Our data indicate that selection and adaptation led to specific patterns of genetic variation in the cultivated tomato germplasm. To date, genomic evidence for the specificity of cultivated tomato landraces has been largely inferred from a limited number of samples or markers. The observed genetic differentiation within contemporary market classes should reflect division into alternative breeding programmes, selection for specific traits (e.g.: fruit shape) and their combinations. Finally, the data indicate that landraces may carry an extended footprint at the genomic level, which deserves further investigation. The disappearance of local varieties represents another cause of reduction of tomato diversity [14, 15, 47] and this study provides evidence to encourage a long-term effort for the characterization and exploitation of cultivated tomato landrace.

## Methods

### Plant material and DNA isolation

The germplasm of cultivated (*Solanum lycopersicum*) and wild tomatoes used in this study is listed in Additional file 1: Table S1. We analysed 214 genotypes which included 30 cherry, 37 fresh-market, 76 landraces and 65 processing accessions of *S. lycopersicum*, along with six wild species. Landraces (also called heritage) tomatoes represent cultivated, open-pollinated accessions that include farmers’ selections and traditional types. Although the exact historical origin of this material is not always known, our landraces can be considered as regional accessions that originated in Italy and whose diversity has been maintained by local farmers. Processing, fresh market and small fruit/cherry varieties represent a selection of commercially relevant cultivars. The classification in different market-classes reflects that of the tomato seed companies. ‘Microtom’, a variety developed for ornamental purposes [48], was included in the cherry group. The analyzed collection included Heinz 1706 and LA 1589, whose genomes have recently been sequenced. DNA isolation was carried out on young true leaves, according to previously reported procedures [49].

### Genotyping

We used the Illumina Golden-Gate assay for large-scale SNP validation, utilizing a customized design based on the 384-format Genotyping Assay. The SNPs’ set comprised polymorphisms, distributed throughout the genome, selected from literature [6, 50] and the SOL Genomics Network (http://solgenomics.net). Briefly, the sequence of each selected locus, including the polymorphic nucleotide and a 60-bp flanking sequence, was submitted to the Illumina Assay Design Tool (Illumina). The GoldenGate assay was arrayed on the BeadXpress Reader (an automated fluidics and multi-laser imaging device platform) using the VeraCode technology (Illumina). The labeled allele-specific PCR products were hybridized to the VeraCode beads, each bearing a locus-specific barcode via the corresponding Illumicode sequence. A supervised allele calling for each locus was accomplished based on the data generated by the GenomeStudio Data Analysis software (Illumina). We tested 192 SNPs. Fifteen were removed from the genetic analysis because of the percentage of missing data points (> 5%). The genotyping with the Illumina GoldenGate platform was carried out at the Parco Tecnologico Padano (http://www.tecnoparco.org/).

### Classification of markers

To determine the physical positions of the SNP markers used in this study, the sequences used to develop these SNPs were Blasted (BlastN) against the tomato genome. Only the top hits with an e-value ≤ 1e-10 were considered. Information on the location of the SNPs and their gene feature details are presented in the Additional file 12: Table S11. Using the available genome annotations (Sl2.40), we categorized the SNPs in “coding” (i.e.: those located in exonic regions) and “non coding” (i.e.: those located in introns as well as intergenic regions). Location in gene models was identified using the SGN genome browser (ITAG2.3 genomic annotation).

### Data analysis

Gene diversity, Polymorphic information Content (PIC), allele frequencies and allelic richness were calculated as already described [51–53] using the PowerMarker [54] and the MSA [55] software. Population differentiation tests and related statistics were carried by PowerMarker as previously reported [56]. Possible population structure was estimated using a model-based Bayesan procedure implemented in the software Structure v2.3 [31] and Structure Harvester [57]. The analysis was carried out using a burning period of 25,000 iterations and a run length of 500,000 MCMC replications. We tested a continuous series of Ks, from 1 to 12, in ten independent runs. We did not introduce prior knowledge about the population of origin and assumed correlated allele frequencies and admixture [58]. The most informative K was identified using the ad hoc statistic ΔK, which is based on the rate of change in the log probability of data between successive K values [59] and the analysis of variance of the log likelihood values using the non-parametric Kruskal–Wallis test [60] (SPSS Statistics 20; IBM). The estimated cluster membership coefficient matrices of the ten runs were permuted so that all replicates have the closest match possible and then averaged across replicates using the Greedy algorithm of the software CLUMMP [61]. To validate the predefined or the estimated population structure, we calculated pairwise Fst and Nei’s standard genetic distance (Dst) between populations [51, 62] using MSA [55]. The reference distribution for P-value calculation of the Fst analysis was based on 10,000 permutations. We identified loci under positive selection between pre-defined populations of cultivated tomato using an Fst-outlier detection method [42] implemented in the software Lositan [63]. We ran 100.000 iterations, using a 0.95 confidence interval and an infinite allele model. Loci that deviate from the expected distribution of neutral markers were identified on the basis of excessively high or low Fst.

## Electronic supplementary material

Additional file 1: Table S1: List of the genotypes analyzed in this study and their classification. (DOCX 24 KB)

Additional file 2: Table S2: SNP Summary Statistics (214 genotype). (XLSX 22 KB)

Additional file 3: Table S3: SNP Summary Statistics in S. lycopersium (208 genotypes). (XLSX 31 KB)

Additional file 4: Table S4: Allele frequency. (XLSX 41 KB)

Additional file 5: Table S5: Allele frequency in the predefined *S. lycopersium* subpopulations. (XLSX 25 KB)

Additional file 6: Table S6: Statistical significance of the pairwise analysis of the allelic richness per locus in the predefined *S. lycopersicum* groups. (XLSX 9 KB)

Additional file 7: Figure S1: Estimation of the optimum number of clusters. Estimation of the optimum number of clusters of tomato accessions according to the analysis of variance of the log-likelihood values (**A**: all SNPs; **C**: non coding SNPs; **E**: coding SNPs) and the Evanno’s method (**B**: all SNPs; **D**: non coding SNPs; **F**: coding SNPs). For the analysis of variance, the graphs display the average (± s.d.) of log likelihood values for each K value. For the Evanno’s method, the graph displays the Delta K [mean (lL”(K)l/SD(L(K))] for each K value. (JPEG 723 KB)

Additional file 8: Table S7: Membership coefficients (Q-matrix) of the genotypes based on STRUCTURE and CLUMPP analyses. (XLSX 52 KB)

Additional file 9: Table S8: Pairwise estimates of Fst and Nei’s standard genetic distance (Dst) between predefined groups or between groups of tomato accessions as inferred by the Bayesian analysis implemented in the STRUCTURE software. (XLSX 16 KB)

Additional file 10: Table S9: Heterogeneity in F-Statistics among loci. (XLSX 11 KB)

Additional file 11: Table S10: Candidate loci under positive selection between landraces varieties and the different market-classes of contemporary varieties. (XLSX 10 KB)

Additional file 12: Table S11: Marker names and their genomic information. (XLSX 22 KB)

## Abbreviations

Dst: Nei’s standard genetic distance
LD: Linkage disequilibrium
PIC: Polymorphic information content
SNP: Single nucleotide polymorphism.
