# Supplementary material for: SNP genotyping reveals genetic diversity between cultivated landraces and contemporary varieties of tomato
Source: BMC Genomics. 2013 Nov 27;14(1):835. doi: 10.1186/1471-2164-14-835 (PMC4046682; doi:10.1186/1471-2164-14-835)
Supplement: Supplementary file 1 — Additional file 1: Table S1: List of the genotypes analyzed in this study and their classification. (DOCX 24 KB) [file 12864_2013_5539_MOESM1_ESM.docx]

Supplementary table 1: list of the genotypes analyzed in this study and their classification.

| Genotype | Market Class |
| --- | --- |
| Birba | Cherry |
| Dardo | Cherry |
| Datterino Lodato | Cherry |
| Datterino Semiorto | Cherry |
| Datterino Esa | Cherry |
| Isi 46343 | Cherry |
| Isi 48130 | Cherry |
| Isi 87112 | Cherry |
| Isi 87134 | Cherry |
| Kikko | Cherry |
| Mascalzone | Cherry |
| Microtom | Cherry |
| Miller | Cherry |
| Minidor | Cherry |
| Okidoki | Cherry |
| Ovalino | Cherry |
| Panarea | Cherry |
| Penny | Cherry |
| Pixel | Cherry |
| PS 6395 | Cherry |
| PS 6PUNTO7 | Cherry |
| Quorum | Cherry |
| Red Cherry | Cherry |
| Ronny | Cherry |
| Santa | Cherry |
| Spotter | Cherry |
| Sultana | Cherry |
| Tigro | Cherry |
| Tomito | Cherry |
| Tondino V710097 | Cherry |
| Atermona | Fresh |
| BB Sel-Por | Fresh |
| Carlota | Fresh |
| Carnaby | Fresh |
| Caronte | Fresh |
| Corallo | Fresh |
| Franco | Fresh |
| Goldmar | Fresh |
| Gordon | Fresh |
| Gotico | Fresh |
| Heline | Fresh |
| Hypnos | Fresh |
| Ibr Grapp | Fresh |
| Isi 36612 | Fresh |
| Isi 36629 | Fresh |
| Isi 36670 | Fresh |
| Isi 68281 | Fresh |
| Jordan | Fresh |
| Kively | Fresh |
| Malinche | Fresh |
| Margot | Fresh |
| Marmande | Fresh |
| Mensei | Fresh |
| Minerva | Fresh |
| Momor | Fresh |
| Monalbo | Fresh |
| Motelle | Fresh |
| PS18 3 2693 | Fresh |
| Rhodia | Fresh |
| Scilla | Fresh |
| Stevens | Fresh |
| Stratos | Fresh |
| Templar | Fresh |
| Teodoro | Fresh |
| Trinity | Fresh |
| Tropic | Fresh |
| Vidal LSL | Fresh |
| Agro/Nocerino 13 | Landrace |
| Agro/Nocerino 14 | Landrace |
| Agro/Nocerino 16 | Landrace |
| Agro/Nocerino 18 | Landrace |
| Agro/Nocerino 19 | Landrace |
| Agro/Nocerino 2 | Landrace |
| Agro/Nocerino 21 | Landrace |
| Agro/Nocerino 22 | Landrace |
| Agro/Nocerino 3 | Landrace |
| Agro/Nocerino 4 | Landrace |
| Agro/Nocerino 5 | Landrace |
| Agro/Nocerino 6 | Landrace |
| Agro/Nocerino 7 | Landrace |
| Agro/Nocerino 8 | Landrace |
| Agro/Nocerino 9 | Landrace |
| Area Vesuviana 14 | Landrace |
| Area Vesuviana 31 | Landrace |
| Casarbore | Landrace |
| Corbara 1:6 | Landrace |
| Corbara 2:7 | Landrace |
| Ercolano 2 | Landrace |
| Etnico | Landrace |
| Faino | Landrace |
| Fiaschetto Determinato | Landrace |
| Gia Giu | Landrace |
| Giallo | Landrace |
| Grottaminarda | Landrace |
| Lampadina Determinato | Landrace |
| Liguria | Landrace |
| Lungo 1 | Landrace |
| Maiese | Landrace |
| Mercato San Severino | Landrace |
| Nocera Inferiore | Landrace |
| Onta | Landrace |
| Padanaro Indeterminato | Landrace |
| Parminatella | Landrace |
| Pisanello | Landrace |
| Pomodorino Vesuvio | Landrace |
| Pomodoro a forma di oliva | Landrace |
| Pomodoro Belmonte | Landrace |
| Pomodoro Nano | Landrace |
| Pomodoro Sorrento | Landrace |
| Ponderosa Indeterminato | Landrace |
| Principe Borghese Determinato | Landrace |
| Principe Borghese Indeterminato | Landrace |
| Roma | Landrace |
| Rosa di Sorrento 10PL | Landrace |
| Rosa di Sorrento ML | Landrace |
| San Marzano 37 | Landrace |
| San Marzano batta | Landrace |
| San Marzano cilindrico3 | Landrace |
| San Marzano cilindrico4 | Landrace |
| San Marzano Schifano | Landrace |
| San Marzano Morini | Landrace |
| San Marzano Murano | Landrace |
| San Marzano Nano | Landrace |
| Sarno 1 | Landrace |
| Scafati 1 | Landrace |
| SMEC20 San Marzano | Landrace |
| Sorrento Adg | Landrace |
| Sorrento Art | Landrace |
| Sorrento Globoso Rosato Indeterminato | Landrace |
| Sorrento Indeterminato | Landrace |
| Sorrento Tondo Liscio Rosato | Landrace |
| Spongillo | Landrace |
| Tondino | Landrace |
| Tondino Determinato | Landrace |
| Tondino Indeterminato | Landrace |
| Tondino Zagarito | Landrace |
| Tondo 1 | Landrace |
| Tondo Liscio Indeterminato | Landrace |
| Ventura Determinato | Landrace |
| Vesuvio 2001 | Landrace |
| Vesuvio Foglia Oliva | Landrace |
| Vesuvio Foglia Riccia | Landrace |
| Vesuvio Pizzo | Landrace |
| Anita | Processing |
| Auspicio | Processing |
| Cancello | Processing |
| Carioca | Processing |
| Cayambe | Processing |
| Cirio3 | Processing |
| Cohiba | Processing |
| Coronel | Processing |
| Decio | Processing |
| Defender | Processing |
| Diaz | Processing |
| Discovery | Processing |
| Docet | Processing |
| Elba | Processing |
| Elgon | Processing |
| Ercole | Processing |
| Everton | Processing |
| Galeon | Processing |
| Genius | Processing |
| Guardalete | Processing |
| Gulliver | Processing |
| Heinz 1706 | Processing |
| Herdon | Processing |
| ISI 17548 | Processing |
| Isi 26761 | Processing |
| ISI 27163 | Processing |
| ISI 36657 | Processing |
| Jet | Processing |
| Joel | Processing |
| Leader | Processing |
| Logan | Processing |
| M82 | Processing |
| Montericco | Processing |
| Ovidio | Processing |
| Pavia | Processing |
| Perfectpeel | Processing |
| Player | Processing |
| Power | Processing |
| Progress | Processing |
| PS 1398 | Processing |
| PS02325446 | Processing |
| PS02326502 | Processing |
| PS02431185 | Processing |
| PS1296 | Processing |
| PS3389 | Processing |
| PS513 | Processing |
| PS8609 | Processing |
| PS9023 | Processing |
| Pullrex | Processing |
| Record | Processing |
| Red Setter | Processing |
| Regent | Processing |
| Remo ibrido | Processing |
| Rio grande | Processing |
| Scipio | Processing |
| Smart | Processing |
| Suerte | Processing |
| TA 209 | Processing |
| Talent | Processing |
| Terranova | Processing |
| Uc 105 j | Processing |
| UC82 | Processing |
| Vegas | Processing |
| Vespro | Processing |
| Vulcan | Processing |
| S. chmielewskii LA 1327 | Wild |
| S. habrochaites LA 1777 | Wild |
| S. lycopersicoides LA2951 | Wild |
| S. neorickii LA 2133 | Wild |
| S. pennelli LA716 | Wild |
| S. pimpinellifolium LA1589 | Wild |
