# Supplementary figures and images for: SNP genotyping reveals genetic diversity between cultivated landraces and contemporary varieties of tomato
Source: BMC Genomics. 2013 Nov 27;14(1):835. doi: 10.1186/1471-2164-14-835 (PMC4046682; doi:10.1186/1471-2164-14-835)

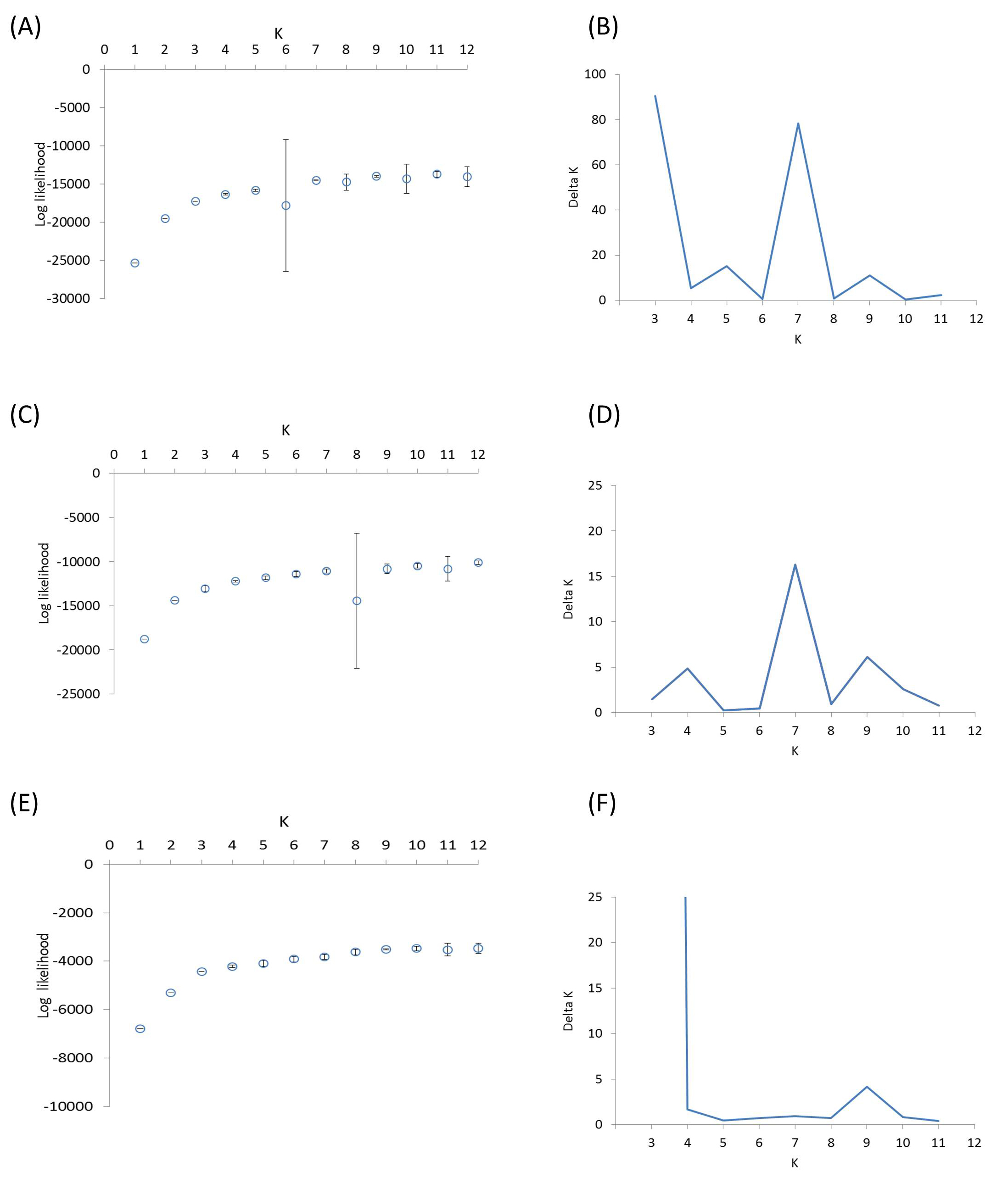

Supplement: Supplementary file 7 — Additional file 7: Figure S1: Estimation of the optimum number of clusters. Estimation of the optimum number of clusters of tomato accessions according to the analysis of variance of the log-likelihood values (A: all SNPs; C: non coding SNPs; E: coding SNPs) and the Evanno’s method (B: all SNPs; D: non coding SNPs; F: coding SNPs). For the analysis of variance, the graphs display the average (± s.d.) of log likelihood values for each K value. For the Evanno’s method, the graph displays the Delta K [mean (lL”(K)l/SD(L(K))] for each K value. (JPEG 723 KB) [file 12864_2013_5539_MOESM7_ESM.jpeg]
